# Generative models and synthetic data in clinical prediction models: Promoting consistency, reproducibility, and transparency

**DOI:** 10.1017/cts.2026.10729

**Published:** 2026-03-25

**Authors:** Anthony A. Mangino, Taha Ahmed, Vincent L. Sorrell

**Affiliations:** 1 Department of Biostatistics, University of Kentucky, USA; 2 Emory University, USA; 3 Department of Internal Medicine, University of Kentucky, USA

**Keywords:** Generative modeling, clinical prediction models, consistency, reproducibility, transparency

## Abstract

**Introduction:**

Reproducibility, consistency, and transparency are essential to responsible and ethical scientific inquiry, though practices supporting these qualities are often neglected. However, in many cases data are confidential or otherwise unable to be shared publicly. This tutorial describes a method utilizing generative adversarial networks (GANs) to create synthetic data that are sufficiently similar to the original dataset in such cases where the source data cannot be shared or where the source data are too sparse as to internally validate results.

**Methods:**

Utilizing an exemplar study that aimed to create a clinical prediction model employing a novel echocardiographic measurement to differentiate between acute coronary syndrome and Takotsubo syndrome, we demonstrate the procedure of fitting a GAN and evaluating the resulting synthetic dataset against the results from the source dataset using conventional analytic methodologies. Further, we include relevant R code and output from this process to aid in implementation.

**Results:**

The procedure we detail yielded a synthetic dataset that was largely similar to the source data used in univariate descriptive statistics, significance testing comparing variables across datasets, data visualizations, and yielded largely comparable secondary model fit and accuracy metrics.

**Conclusions:**

We demonstrated that through the implementation of a well-tuned GAN, synthetic data can be generated as a sufficiently faithful simulacrum of the source data for the purposes of internal validation, transparency of method, and reproducibility of analytic results.

## Introduction

The replication crisis is a well-documented condition across a variety of empirical domains, ranging from psychology [1] to public health and clinical science [2,3], among others. Simultaneously, the National Institutes of Health (NIH) have implemented a Data Management and Sharing Policy, effective January 25, 2023 (NOT-OD-21-013) [4] to facilitate transparency and external accountability to the broader scientific community. However, data may not be sufficiently plentiful to facilitate reproduction of the original results, nor permitted to be shared due to ethical, legal, or confidentiality obligations. The question becomes how reproducibility and transparency might be promoted given these practical constraints. To demonstrate the utility of one such generative modeling (GM) technique and synthetic data in promoting reproducibility and transparency, we describe the procedure of reproducing and validating results from a novel clinical prediction model to aid in diagnosis of Takotsubo syndrome (TTS) in a small, unbalanced sample of patients.

### Purpose and use of GM techniques

Of principal interest, the use of GMs and synthetic data allow for four major purposes:

- Assessing and ensuring stability of primary model results and fit;
- Reproducing results from a comparable dataset without sharing sensitive or confidential data (e.g., patient records);
- Balancing datasets where the outcome and/or treatment groups are otherwise unbalanced; and
- Ensuring transparency of study procedures and analytic methods.

One area in which reproducibility and transparency are of paramount importance is clinical prediction modeling, which compiles a wide variety of data to arrive at diagnostic, prognostic, and treatment decisions [5]. Presently, we examine the use of classification modeling in which a discrete number of outcomes is possible [6]. Two notable problems exist in this context: the number of cases in the sample may be small, and/or one of the outcomes may be far less common than another. This phenomenon of unbalanced outcomes is common in situations of diseases such as Maturity-Onset diabetes of the Young [7], graft-versus-host disease [8], or in the present example, TTS [9] in which the number of individuals in the focal group is generally much smaller than those diagnosed with more common cardiac conditions. In such cases, Eisenstein [10] notes that while classification models may achieve high overall accuracy rates they will be ineffective in correctly identifying members of the smaller group. Consequently, classifiers correctly predicting the larger group are only minimally useful. These unbalanced outcome groups, overall small sample size, and limited ability to perform relevant subgroup analyses adversely impact reproduction and stability of results, and often prevent data sharing due to small cell sizes and threats to patient confidentiality.

With respect to data balancing, several data methods – including synthetic minority oversampling technique (SMOTE [11]), random oversampling examples (ROSE [12]), and bootstrapped minority oversampling [13] – have been used to balance outcome groups; SMOTE, in particular, uses synthetic cases, though only those that would likely be found within the existing sample. However, these methods are relatively purpose-specific and have a limited range of application. The family of GM techniques mark a substantial contribution to the machine learning (ML) literature to create synthetic data, particularly with the advent of generative adversarial networks (GANs) by Goodfellow [14]. These methods have gained widespread implementation in contexts including image processing [15,16] and the generation of believable synthetic patient records [17].

Data privacy and security are another salient benefit of constructing *ad hoc* generative models as oftentimes, online large language models (such as ChatGPT or Copilot) may not meet security standards of academic institutions, corporations, or governmental organizations. The operative element of ensuring data privacy is the ability of generative models to create synthetic data that sufficiently closely represent the topography and relationships of the source data while reducing, though not eliminating, the risk of identification or direct copying of actual cases. Of recent note is the development and implementation of “authenticity” and “precision” metrics by Alaa et al. [18] in which it was demonstrated that well-tuned generative models (specifically GANs) yielded synthetic data that were sufficiently similar to the source data to yield highly accurate results in a prediction model, but with cases that were appreciably distinct from the source data. Similar results were found by Endres et al. [19] using a “proximity” metric with GANs and SMOTE, among other GM frameworks. Additionally, while El Emam et al. [20] recommended the use of multiple synthetic datasets (a practice with which we agree), they identified that even in the case of single-dataset use, membership disclosure (a relative F1 score constructed to compare the correct patient-level treatment group identification with versus without using synthetic data) was acceptably low, defined as a relative F1 score less than 0.2, which was met by all datasets in their investigation (range = 0.0035–0.0805).

While the similarity metrics discussed may be useful in assessing dataset quality, Beaulieu-Jones et al. [21] assert that differential privacy, specifically, must be ‘mathematically provable.’ The authors detail a GAN architecture in which the effect of any single case was limited, and random noise proportionate to the impact the case had was added. Their approach provides quantifiable privacy guarantees (ϵ, δ), where ϵ bounds how much the inclusion or exclusion of any single case can affect the probability distribution of synthetic outputs, and δ represents the probability that this guarantee does not hold. At present, while Beaulieu-Jones et al. have established a Python implementation of their GAN architecture, including privacy metrics, such an implementation is not readily available in the R Statistical Software package at the time of this writing. Therefore, we do not recommend sharing synthetic data as we demonstrate generating it until such a time where formal privacy metrics can be implemented and assessed. Given these purposes, the principal goal in presenting this applied example using an existing study predicting TTS using synthetic data is to a), ensure consistency of results obtained from the source data in Ahmed et al.’s [9] original study, which was conducted on an otherwise limited sample and with little opportunity to expand the analytic sample, and b) provide a concrete workflow for researchers to implement in their own work such that transparency, reproducibility, and generalizability of the methods of inquiry may be demonstrated and enhanced, thus improving the overall quality of a study or analysis sequence.

Not every study design, research question, dataset, and context is conducive to the implementation of GM techniques and synthetic data. Consequently, several practical questions must first be sufficiently addressed to ensure that utilizing these techniques is appropriate. To aid the reader in determining whether synthetic data (and generative models, largely) are appropriate, we provide several questions we employed in our inquiry in Table 1 alongside our responses to these questions.

**Table 1.** Guiding questions prior to implementing generative models and synthetic data

Question	Example
Is my purpose clear? Am I intending to use synthetic data for sharing? For internal replication? For enhancing a prediction model? Or another purpose?	The synthetic data will be used to train a prediction model on an otherwise small sample from a limited patient population, and internally validate the prediction accuracy metrics obtained from the source data using the synthetic data.
Are my data well-suited to use in a GAN? Do I have complete data with simple relationships? Or a large, complex dataset with numerous missing data patterns?	This is a small, complete dataset with very clear and simple relationships among variables. Missing data are not present in any variables used in the prediction model.
Have I properly tuned my model’s hyperparameters? Am I forcing my GAN to overfit or underfit? Am I using an optimization method to tune the GAN?	The GAN was tuned using a grid search procedure with the final specification yielding both the lowest loss value and the most sensible descriptive statistics and bivariate relationships among variables across the entire grid.

GAN = generative adversarial networks.

### The architecture of GANs

GANs are constructed as two neural networks – a generator (G) and discriminator (D) – competing with one another in a “minimax game” for each to attempt to outperform the other [14,22]. A GAN utilizes these neural networks working in tandem as follows:

- The G network is provided with a randomly generated matrix of values and attempts to create synthetic cases that mimic the cases in the source dataset.
- The D network then receives a minibatch (a subset of the full dataset) from both the generated and source datasets, and must determine whether each case is real or fake.
- Error information from the decisions made by D is then fed back to G, which updates its parameters in order to generate a new set of cases to be passed again to D.
- This process of successive refinement of both D and G is repeated across a high number of epochs (training cycles) such that an optimum is attained where the accuracy of G is maximized and the accuracy of D is minimized.

**Figure 1.**
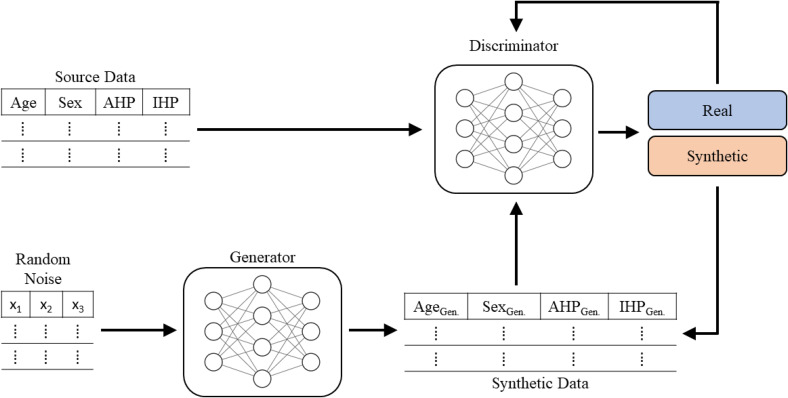
GAN architecture and training process. GAN = generative adversarial networks.

This process is shown below in Figure 1.

Early studies using GANs focused on image generation and demonstrated them as capable of creating synthetic images closely resembling source images and often with greater clarity [14–16]. Limited research has yet been done on GANs using the two-dimensional “flat file” format of most electronic health record (EHR) databases. Choi et al. [17] instantiated a GAN architecture incorporating autoencoders (GMs specializing in the deconstruction, representation, and reconstruction of data; med*GANs*) for the purpose of creating synthetic patient records. The results of Choi et al.’s [17] study showed that *medGAN* yielded cases closely resembling source patient files. Further, Mangino et al. [23] employed GANs as a method of both balancing groups in a prediction modeling context and enhancing the prediction of adolescents at risk of attempting suicide in the 2017 Youth Risk Behavior Survey. Mangino et al.’s study also illustrated that outcome group proportions differed: the source dataset had an approximate 10:1 ratio of adolescents who had versus had not attempted suicide within the last year, and the synthetic data was far more balanced with an approximate 10:13 ratio in these groups. Despite documented differences, both Choi et al. [17] and Mangino et al. [22] demonstrated the utility and efficacy of GAN models for use in two-dimensional data structures, as typically seen in EHR databases.

In studies of rare diagnoses with a goal of constructing a prediction model, both unbalanced outcomes and an overall small sample may manifest. In both cases, prediction models may be drastically underpowered, have limited generalizability, or may simply not be able to accurately capture the relationships among predictors of the disease and the disease itself. The result is a limited ability of such prediction models to be applied in clinical contexts or extended to different patient populations. The ability of a well-tuned GAN to craft a synthetic dataset that closely matches univariate and multivariate distributions and relationships may provide ample opportunity to train secondary models (i.e., those intended for interpretation or prediction) with larger samples for validation with real data, or to provide shareable datasets accompanying manuscripts where sharing source data is disallowed.

A further area in which GANs may have a substantial contribution is in the stability and internal consistency of analyses, particularly in studies with small samples. Bree [24] explains this issue of stability as being partially dependent upon minute input errors in smaller sample sizes and conventional parametric models, specifically when collinearity may be present (common in models designed to maximize predictive accuracy). Yu [25] discusses methods including the bootstrap, regularization, and cross-validation as methods for aiding in stability of model parameter estimates, each with broad application. However, the limitations of these methods are the same: the source data are rote duplicated and perturbed in a finite number of ways. Models or data may be slightly perturbed (e.g., robust/nonparametric models or bootstrapping), but the same cases present in the source data are the only cases possible in the perturbed data. Similarly, data balancing methods (e.g., SMOTE) are more purpose-specific, and while they can create sufficiently well-perturbed synthetic data, they are not flexible, design-agnostic methodologies. Alternatively, GANs may provide cases sufficiently outside the confines of the source data to test the stability of secondary models without straying egregiously from the behavior of the source data or being conditional on the study design or data type [18–20].

### Validating a prediction model for TTS: background

The following exemplar is based on the Two-Chamber Apical Kinesis Observation (TAKO) study [9], which crafted a clinical prediction model for differentiating a TTS diagnosis from the more common left anterior descending coronary artery syndrome (LAD-ACS, henceforth simply ACS).

Briefly, TTS (colloquially “broken heart syndrome”) is a chest pain syndrome characterized by transient left ventricular dysfunction in the absence of significant epicardial coronary artery stenosis, distinguishing it from typical myocardial infarction. Oftentimes, TTS is preceded by a substantial physically or emotionally stressful event triggering classic ventricular regional wall motion abnormalities [26] and is often mistaken for ACS due to its overlapping clinical presentation. Following patient presentation to the emergency room, it is typical for clinicians to use coronary angiography to diagnose whether the coronary event was TTS, ACS, or a different disease mechanism entirely. However, angiography is an invasive method requiring arterial puncture for access and catheter engagement to visualize the coronary arteries. While it typically reveals reduced or no blood flow in ACS, TTS patients usually demonstrate normal or near-normal coronary flow, helping differentiate the two conditions. As a potential solution, Ahmed et al. [9] proposed a noninvasive, reliable, and easily deployed method using transthoracic echocardiography (TTE) to indicate whether the given chest pain event was ACS or TTS. Measurements of endocardial hinge points – the point of transition from normal to abnormal regional wall thickness – were obtained during heart contraction (in millimeters), with the central two measurements being the anterior hinge point (AHP) and inferior hinge point (IHP) relative to the mitral valve annulus. The researchers further proposed that the ratio of the hinge point measurements, the AHP/IHP ratio, contingent on patient sex (and the associated difference in heart size) would allow for a valuable predictive marker for differentiating between TTS and ACS cases.

The initial cohort with complete data included 52 patients with TTS (46 female [88.5%]) and 50 patients with ACS (20 female [40%]) aged 22 to 84 presenting to University of Kentucky Healthcare between January 2010 and December 2021 with evidence of left ventricular apical hypo-, a-, or dyskinesis who also received both a TTE and coronary angiogram within 72 hours of presentation. The first phase of Ahmed et al.’s study involved two clinicians evaluating the TTEs to record the AHP and IHP measurements, then constructing a logistic regression model utilizing the AHP/IHP ratio and patient sex to predict the charted diagnosis of either TTS or ACS. The model was specified as:

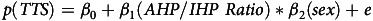

such that the interaction of AHP/IHP ratio and patient sex yielded separate AHP/IHP ratio cutoffs for a TTS diagnosis for male and female patients in the sample. This phase of the study yielded accuracy rates of approximately 84.85% for men with an AHP/IHP ratio cutoff of 0.84 and 91.67% for women with an AHP/IHP ratio cutoff of 0.96; the model had an AUC of 0.948.

Ahmed et al.’s study [9] continued to evaluate the model with an internal validation sample (the same cases with measurements taken and diagnoses rendered by different clinician-evaluators) with limited results, thus indicating that additional predictors and/or additional training of the readers of the validation cohort cases may be beneficial in ensuring this model could be deployed in a clinical context. Our focus in this illustration, however, is the method by which this limited sample of 102 patients may be used to generate synthetic data for each of the purposes we initially described. Further, while metrics exist for quantifying the similarity of two datasets, such metrics may as yet be unfamiliar to many non-statisticians, so while we recommend examining these measures (see Alaa et al. [18], Emam et al. [19], and Endres et al. [20] for specifics), we instead examine more commonly known measures including Cohen’s d and Cramer’s V effect sizes, odds ratios (ORs), and model fit and accuracy statistics.

### Validating a prediction model for TTS: synthetic data generation and examination

Though GANs are implemented in a variety of software packages, the present exemplar utilizes the R Statistical Software Package [27] and the RGAN package [28] due to the ease of use, ready availability, clear documentation, and current development cycle. We provide a singular R script and synthetic dataset for the interested reader to follow this procedure and incorporate it into their own workflow and study design at: https://github.com/UKBiostatCIRCL/Public-Code/tree/main/GAN%20Tutorial%20Manuscript.

The process of constructing and evaluating the GAN follows five steps:

1. Create and fit a transformer to the source data to standardize continuous variables and one-hot encode categorical variables (i.e., create separate binary-coded columns for each category within categorical variables).
2. Specify the constituent G and D network architectures, including number of hidden layers/nodes, activation functions, loss functions, etc.
3. Fit the GAN to the source data, assessing data generation periodically.
4. Evaluate the final synthetic dataset and resulting secondary models to determine the extent of similarity to the source data.
5. Repeat steps 2-4 as necessary to ensure sufficient – but not excessive – similarity to the source data (i.e., generative ML models can overfit their training data).

Prior to beginning the workflow, data must be cleaned to ensure:

- The dataset is a matrix, array, or dataframe object;
- Continuous variables are numeric or integer type;
- Categorical variables are numeric, integer, or factor type and numerically coded.

**Figure 2.**
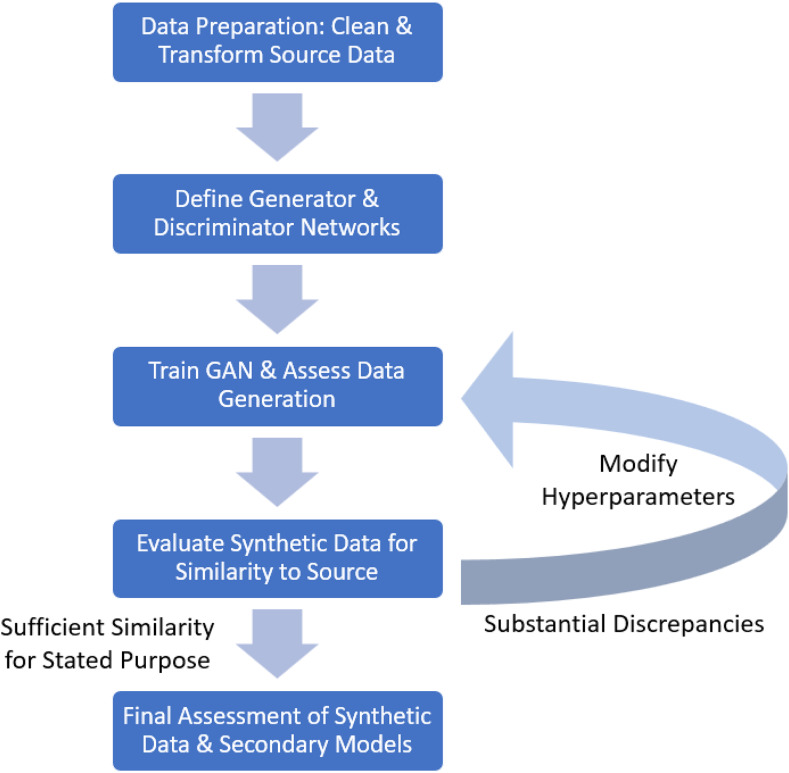
Flowchart of creating and evaluating synthetic data.

Data cleaning process should also involve only retaining those variables believed to be relevant, though it has yet to be determined the extent to which omitting variables ancillary to the research question may affect the GAN estimation process. Missing data cannot be present in the source dataset, which we note as a limitation of this method in inquiries using EHR data. There are GAN architectures for imputing missing data, though these are beyond the scope of this demonstration (see Yoon et al. [29] or Alharbi and Kimura [30]). We encapsulate this process in the flowchart in Figure 2.

### Step 1: fit the transformer to standardize source data

In this step, the transformer standardizes continuous variables to have a mean of 0 and standard deviation of 1, and one-hot encodes categorical variables (creating one binarized column per category). The process of transforming the data allows for the GAN to more easily learn the underlying relationships within the dataset, after which the transformer can back-transform the synthetic data into the source data format and scales.

### Step 2: specify G and D network architectures

The RGAN package has several required hyperparameter specifications. Briefly, *parameters* are values models learn through the estimation process that quantify associations between variables (e.g., coefficients); *hyperparameters* are user-defined values set prior to the training process of a model with the intention of controlling how the training process proceeds (e.g., minibatch size). Few rules exist for determining the optimal hyperparameters for any single neural network, much less a GAN, though we found the default architecture from the RGAN package works well, or may benefit from only minor modifications. For the interested reader, we recommend Hagan’s [31] or Aggarwal’s [32] texts on specifying and evaluating neural networks.

We employed the default hyperparameters, which specified the discriminator and generator to each be dense networks with two hidden layers, 64 nodes, and a dropout rate of 0.5; the generator also had five noise dimensions (the random noise shown in Figure 1). While this step is an integral component of fitting the GAN, the sheer volume of different architectures makes this a step worthy of careful consideration on its own.

### Step 3: fitting the GAN to source data and extracting synthetic cases

Because every dataset is constructed differently and contains different relationships among the variables, so too must the hyperparameters for the GAN differ to account for the source dataset’s idiosyncrasies. In a typical ML pipeline, a grid search algorithm is commonly used to identify the optimal hyperparameters by specifying a list of values for each hyperparameter, fitting the model with each combination of hyperparameters and obtaining some accuracy or error metric, modifying one hyperparameter value and refitting the model to extract that same accuracy or error metric, and repeating that process for all combinations in the grid. This is often a lengthy, but integral, process.

We utilized the default hyperparameters for the G and D networks, but performed a grid search to identify the optimal GAN hyperparameters with the final list being:

- Noise dimensions = 5,
- Value function = “Wasserstein,”
- Learning rate = 0.0001,
- Two time-scale update rule (TTUR) factor = 8 (this value ensures the learning rate of the generator is lower than that of the discriminator to avoid “mode collapse” in which all outputs are the same value),
- Minibatch size = 30,
- Epochs = 400,
- Number of synthetic cases to be generated = 1020 (n_TTS_ = 102 × 10).

Our grid is illustrated in Table 2 and the full grid search procedure is included in the accompanying R code. The loss metric used to determine the optimal set of hyperparameters was the Wasserstein critic (discriminator) loss function [33] averaged over the last 20% of epochs in order to ensure stability and minimize bias in any final epoch due to stochasticity. An additional layer of graphical evaluation – in the present case, assessing the scatterplot of the AHP and IHP measurements for the source and synthetic cases – is advised during the data generation process (DGP); often, graphical evaluation can provide insight into the behavior of the synthetic data at various epochs to aid in determining hyperparameter specification and final data evaluation.

**Table 2.** Hyperparameter grid for GAN training

Hyperparameter	Values tested
Noise dimensions	1, 3, 5, 10
Base learning rate	0.0001, 0.001
TTUR factor	2, 4, 8
Batch size	15, 35, 50, 90
Epochs	250, 400, 500, 1000

GAN = generative adversarial networks.

Once the GAN is trained, synthetic cases are extracted. Synthetic data must then be back-transformed and cleaned to reflect the same format and variable types as the source data.

### Step 4: evaluating synthetic dataset and fitting secondary model

Because each procedure has numerous pieces of evidence to consider, evaluating the dataset and model are discussed separately. At this stage, the synthetic data may be exported for use in alternative statistical software (e.g., .csv format). To maintain consistency, we performed all evaluation in R.

### Evaluating the dataset

An initial examination of evaluating the synthetic data entails first assessing the results of the grid search from Step 3; the final dataset should be synthesized from the model yielding the lowest loss metric. Once this condition is ensured, as is the case with any dataset, an exploratory data analysis procedure is necessary, though with the additional comparison to the source dataset. The source data are assumed to be the “ground truth” to which the synthetic data must closely align. It is common that generated data have properties (e.g., central tendency, cell proportions) that vary appreciably from the source data. However, we recommend taking the evidence of the evaluation process holistically to determine whether the GAN was well-tuned or if additional hyperparameter tuning is necessary. A single piece of evidence – such as an impossible value of a continuous variable – should not be the sole deciding factor in determining whether to retain the synthetic dataset.

We began by extracting univariate descriptive statistics including measures of central tendency and variability, histograms or scatterplots for continuous variables, and frequencies, proportions, and bar charts for categorical variables. This summary should be inspected for any obvious aberrant, impossible, or missing values. Missing values in particular should not exist in synthetic data and indicate a failure in the DGP, typically manifest as entire variables or the entire dataset being empty. For example, our synthetic dataset had negative medical record numbers, and IHP and AHP measurements, all of which should be exclusively positive values. It is important to note that impossible values of individual variables may not be a definitive indication that the GAN has failed, as this is a well-known phenomenon in situations of missing data imputation (aside from predictive mean matching [34]). Oftentimes, imputation models intend to preserve the *relationships* between variables, which may entail impossible values for any one case or variable. The same phenomenon appears as part of the GAN DGP.

Of more interest is the stratified comparison of each variable by data source alongside significance tests to determine whether significant differences exist between the data sources on each variable. In these comparisons, it is not necessary that all tests be non-significant, but rather that the measures of central tendency and variability are not *practically* different, or not sufficiently different as to appear as if the real and generated data came from different populations. For example, if the mean age in the synthetic TTS dataset were 40 years (sd = 4), the comparison with the source data mean age of 59.6 years (sd = 12.7) would indicate an egregious and obvious discrepancy. An effect size measure is advised to accompany the significance test, especially when the combined sample size of the synthetic and source data is large and nearly all tests are statistically significant. The magnitude of difference – assuming use of an appropriate effect size for the data type and distribution – could provide stronger statistical evidence indicating whether the source and synthetic datasets are sufficiently different from one another to be practically different. Table 3 illustrates the results of the comparison between datasets with accompanying Cohen’s d and Cramer’s V effect sizes all indicating small to moderate differences. Significance tests used were t-tests for continuous variables, chi-square tests for categorical variables, and Fisher’s exact test for the Diagnosis by Sex composite variable.

**Table 3.** Descriptive statistics and unadjusted analyses comparing synthetic and source data

	GAN, *n* = 1020	Source, *n* = 102	*p*-Value	Effect size*
**Sex**			0.019	0.070
Female	773 (75.78%)	66 (64.71%)		
Male	247 (24.22%)	36 (35.29%)		
**Age**			0.425	0.109
Mean (SD)	58.54 (9.12)	59.58 (12.72)		
Range	26.87–98.05	22.00–84.00		
**Inferior hinge point relative to mitral** **annulus (cm)**			0.209	0.113
Mean (SD)	4.51 (1.76)	4.71 (1.47)		
Range	−2.49–11.03	2.10–8.70		
**Anterior hinge point relative to mitral** **annulus (cm)**			0.004	0.321
Mean (SD)	3.73 (0.92)	4.02 (1.00)		
Range	−0.90–6.69	2.10–6.60		
**Anterior/inferior hinge point ratio**			0.082	0.186
Mean (SD)	0.92 (0.18)	0.89 (0.18)		
Range	0.30–1.77	0.39–1.43		
**Diagnosis**			0.116	0.047
ACS	588 (57.65%)	50 (49.02%)		
TTS	432 (42.35%)	52 (50.98%)		
**Diagnosis by sex (% of full sample)**			<0.001	0.196
Male, TTS diagnosis	3 (0.29%)	6 (5.88%)		
Male, ACS diagnosis	244 (23.92%)	30 (29.41%)		
Female, TTS diagnosis	429 (42.06%)	46 (45.10%)		
Female, ACS diagnosis	344 (33.73%)	20 (19.61%)		

* Effect sizes used were Cohen’s d for continuous variables and Cramer’s V for categorical variables.

GAN = generative adversarial networks.

We note here that the proportional representation of males with a TTS diagnosis in the synthetic dataset is appreciably smaller than in the source data (0.29% compared to 5.88%; *p* < 0.001, *V* = 0.196). Depending on the intended purpose of the synthetic data, this feature may be acceptable, such as cases where the synthetic data are being shared to illustrate a given analytic method. In cases where a truly faithful representation of the source data is sought, this feature may warrant additional tuning of the GAN or its constituent G and D networks.

In the event that the synthetic data are intended to be isomorphically analogous to the source data, multiple runs of the GAN and accompanying extraction of synthetic cases may be required. In contrast, if single or small groups of cases are sought, the standard may be somewhat relaxed.

### Fitting secondary model

Once the descriptive properties of the synthetic dataset have been evaluated and differences quantified, the secondary model can be fit. We implemented a logistic regression model with the same specification as Ahmed et al.’s study [9] fit to the synthetic data; results from the model fit to the original TTS data are included in Table 3. Note that the ORs are inordinately large for both models and functionally uninterpretable; due to the model’s purpose being for prediction, not explanation, this is not of concern and was not a focus of Ahmed et al.’s study. Further, while the ORs differ by a factor of 6000, neither appear to yield ORs that indicate a different relationship of the AHP/IHP ratio and diagnosis. Secondary model evaluation includes assessing the fit statistics and comparing them to those from the original model, evaluating the within-sample predictions (how well the synthetic-trained model predicts diagnosis within the synthetic data), and evaluating the between-sample predictions (how well the synthetic-trained model predicts diagnosis within the source data).

**Figure 3.**
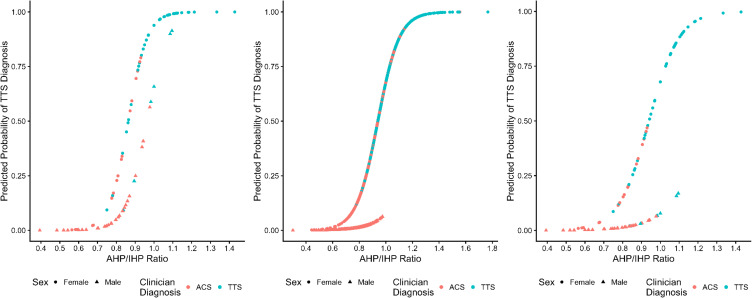
Diagnosis probability plots of logistic regression models trained and evaluated on source and synthetic data. **Left panel** is the source-trained and evaluated model; **middle panel** is the synthetic-trained and evaluated model; **right panel** is the synthetic-trained, source evaluated model.

Key components for interpretation in this example are the model fit statistics (Brier Score, McFadden’s pseudo-*R* ^2^, and AUC), the plot of diagnosis probabilities for each dataset, and the overall accuracy, sensitivity (correct TTS diagnosis predictions), and specificity (correct ACS diagnosis predictions) measures from each model. Figure 3 shows the probability plot for the secondary model predicting diagnosis in the synthetic data in the right panel, and the probability plot for the secondary model predicting diagnosis in the source data in the left panel. Table 4 includes model fit statistics and accuracy metrics for both models as well as the between-sample prediction accuracy metrics. The synthetic-trained model had little uncertainty in the predicted diagnosis and – in conjunction with accuracy metrics – had adequate fit to the synthetic data; overall accuracy was 82.84%, sensitivity was 85.71%, specificity was 78.94%, and the model had an AUC of 0.910 (95% CI: 0.893–0.927), all indicating adequate fit.

**Table 4.** Accuracy metrics, model fit statistics, and model effect sizes for logistic regression

Model fit statistics	Model fit and applied to source data	Model fit and applied to synthetic data	Model fit to synthetic data and applied to source data
Brier score	0.096	0.121	0.141
McFadden’s pseudo *R* ^2^	0.579	0.447	–
**Model prediction accuracy**			
Probability cutpoint	50%	50%	20%
Overall accuracy	85.29%	82.84%	83.33%
Sensitivity	84.00%	85.71%	82.00%
Specificity	86.54%	78.94%	84.62%
AUC (95% CI)	0.948 (0.910, 0.985)	0.910 (0.893, 0.927)	0.936 (0.894, 0.978)
**Model effect sizes**			
**Effect**	**Odds ratio (95% CI)**	**Odds ratio (95% CI)**	–
Baseline odds	0.00 (0.00, 0.00)	0.00 (0.00, 0.00)	
AHP/IHP ratio: Female	484,185,664.51 (328,794.03, 9,487,969,074,313.19)	251,093.72 (42,615.02, 1,708,155.83)	–
AHP/IHP ratio: Male	60,861,987.50 (44,437.46, 878,002,771,199.87)	10,096.94 (808.71, 118,419.12)	–

The source-trained model yielded similarly accuracy with more uncertainty in the probability plot. Overall accuracy was 85.29% with sensitivity being lowered to 84% and specificity remaining at 86.54%. The AUC was still at an appropriate vale of 0.948 (95% CI: 0.910–0.985). Similarly, the synthetic-trained model performed comparably when predicting diagnosis in the source data with an overall accuracy of 83.33%, sensitivity of 82.00%, and specificity of 84.62%; AUC was similar to that of the source-trained and evaluated model at 0.936 (95% CI: 0.894, 0.978). The pseudo *R* ^2^ value was also somewhat lower for the synthetic-trained model at 0.447 compared to the value of 0.579 in the source-trained model. Calibration of model-derived predicted probabilities and actual diagnosis groups was quantified for each scenario using the Brier score, with lower scores indicating greater calibration. The source-trained and evaluated model had a Brier score of 0.096, which somewhat outperformed the synthetic-trained and evaluated model’s score of 0.121 and the synthetic-trained and source tested model’s score of 0.141.

Collectively, these results indicate that the logistic regression model trained on the synthetic data provided only slightly worse fit to the synthetic data compared to the original model trained on source data and yielded similar results when trained on synthetic data and tested on the source data. In such cases as this, it is possible that synthetic data represent a greater amount of variability than the source data and, as a result, may generalize more readily to the validation sample discussed in Ahmed et al.’s original study, though evaluation of this is beyond the scope of this demonstration.

It is precisely in this additional representation of cases that synthetic data may be most useful, as a perfectly isomorphic synthetic dataset would paradoxically inhibit validation of source data results and perhaps prohibit data sharing. It is expected that model fit, accuracy, and/or error metrics, coefficient estimates, and standard errors will differ between secondary models trained and evaluated on source and synthetic data, but when interpreted in conjunction with the univariate descriptive statistics and bivariate data visualizations, the more similar these metrics are to one another, the more likely the synthetic date are to capture the relationships found in the source data. In cases where the secondary model is intended for explanation, not prediction, coefficient estimates and standard errors may be examined in detail, though this was not a key component of the present exemplar due to the uninterpretable large coefficient estimates of both models. We underscore the fact that model fit and accuracy metrics are not sufficient, but the synthetic data themselves must also be evaluated for similarity to the source data. Holistically, the results obtained from our model trained on synthetic data indicate that the results of Ahmed et al.’s [9] original inquiry are, indeed, consistent and warrant external validation.

## Discussion

We have demonstrated the method by which GANs can be used to create synthetic datasets that appear and function similarly to their source dataset, and the method by which this similarity can be quantified and evaluated using readily available and familiar measures that are used in a wide variety of study designs and analysis sequences. While the use of synthetic data is not a novel endeavor, we have demonstrated that it can be used by clinical practitioner-researchers and statisticians unfamiliar with GMs without an extensive amount of technical knowledge of ML pipelines or as-yet unfamiliar similarity metrics, and without jeopardizing patient confidentiality or data security.

Despite the ease of implementation and the ubiquity of online generative AI/ML tools, little rigorous work has been done illustrating the method by which these models can be implemented in an *ad hoc* “in-house” fashion. Our purpose was to illustrate a procedure for this evaluation process in a streamlined and practical fashion distinct from more computationally intensive methods and as-yet novel metrics in the ML literature (e.g., similarity metrics, various loss metrics). Effective use of GMs requires as firm and thorough understanding of the data source, research questions, and relationships among variables as any other model and should be treated with a commensurate amount of deference.

Of principal interest is the output of the GAN implementation and resulting synthetic dataset and secondary models discussed in the final section of the tutorial. It was demonstrated that while the synthetic data were similar to the source data, the similarity depended heavily on which metric was used: results differed by univariable and bivariate comparisons, and model accuracy metrics. In the case of the relative fit statistics AIC and BIC, it was evident that the source data provided better model fit than did the synthetic data. However, the raw accuracy, sensitivity, and specificity metrics did not differ substantially. Through the process of tuning the GAN hyperparameters for this demonstration, raw accuracy metrics fluctuated, sometimes being higher or lower than those from the model trained on the source data; model fit statistics largely remained similar, often with substantially higher AIC and BIC statistics and somewhat lower McFadden pseudo-*R* ^2^ values. Despite higher accuracy metrics, the univariate distributions and bivariate associations often changed dramatically from the source data. The hyperparameters chosen for this demonstration represent the best balance between sufficiently similar univariate descriptive statistics, bivariate relationship topography and magnitude, model fit statistics, and raw accuracy metrics.

In the interest of reproducibility, transparency, and consistency, we acknowledge that the use of synthetic data is not the sole remedy to the limitations or absence of these qualities in empirical inquiry. However, far too often data sources are not shared, analytic results are not reproduced, and results are not validated by researchers internal or external to the team due, in part, to the inability of researchers to share their source data or obtain an appropriate validation sample. Similarly, analytic methods and results are often limited to the narrative in the “Methods” sections of manuscripts with software scripts and data sources not shared. Utilizing GANs to create a shareable simulacrum of these confidential source data may provide additional context and support to research teams intending to verify their own results with similar data, and external researchers in reproducing the analytic methods and results of published studies. It is our hope this exemplar may catalyze additional data sharing and reproduction of primary results.

The potential utility of GM techniques – not exclusively GANs – is vast, and the principal contribution to the literature is incorporation of this methodology into study designs and workflows for different purposes, with different data sources and variable types, and using different secondary models (e.g., random forests instead of logistic regression). This demonstration has been used due to the exemplar dataset being small, complete, with relatively simple variable types and distributions, and with an established and easily understood secondary model. Therefore, results may vary greatly depending on the data source and modeling frameworks employed. Additionally, we did assess different hyperparameter specifications through grid search and found additional tuning to contribute substantially to the efficacy of the GAN. For the purposes of this tutorial, we evaluated results through the procedure described in this tutorial to obtain a “typical” representation of synthetic data for the TTS source data. Future research will thoroughly assess the effects of hyperparameter tuning on tasks including secondary model parameter recovery and fit as well as univariate and multivariate measures of central tendency, dispersion, and frequency.

Despite the complexity of GMs, our goal was to illustrate their utility to biomedical researchers as a practical tool rather than a novelty, their responsible and ethical use, and the ease of implementation. This tutorial is intended as an introduction to this workflow, and using the R script we provide could serve as an initial training opportunity to test for the interested reader’s own work. While AI tools (e.g., ChatGPT) have gained a majority share of the GM domain, the underlying modeling architectures have not been used to their fullest potential in applied research. However, as qualities of transparency, reproducibility, and generalizability of results comes to the forefront of the conversation among scientists and the general public, it is essential that GMs not be relegated to a strictly commodified productivity tool, but rather as a method by which scientists can address these necessary qualities in scientific inquiry.

### Limitations

Several caveats and future examinations must be addressed. As is the case with any discriminative model, such as a logistic regression, the choice of predictor variables to include is paramount. Little is known about the behavior of GANs when datasets include or forego ancillary or “noise” variables that are not otherwise planned for inclusion in a secondary model. In the present case, it is possible that variables such as stenosis percentage, troponin values, or body mass index may be relevant covariates in a regression model and in the GAN DGP. Carefully considering the causal chain or directed acyclic graph is requisite not only for a well-conceived secondary model, but perhaps also for an optimally-performing GAN. Additionally, because the GAN functions as a secondary DGP – separate from that of the source data – it is possible that this secondary DGP introduces second-order bias. This is as yet an area of limited study. Similarly, in cases with missing data, we have established that the currently available GAN architectures cannot handle missing data (aside from those constructed for imputation), therefore any biases or discrepancies generated by the imputation procedure may be either exacerbated or tempered by the GAN DGP.

An additional limitation of this exemplar is the use of only a single synthetic dataset. While the synthetic dataset was ten times larger than the source data, it was nonetheless only drawn as a single output from the GAN. El Emam et al. [20] recommend that multiple (up to ten) synthetic datasets be used and results from secondary models averaged across the datasets in a manner not unlike pooling of results in an imputation context. This is a practice with which we agree given the stochasticity built into any singular run of a GAN. Similarly, because readily available implementation of differentially private GANs with formal privacy guarantees (ϵ, δ) are not yet available in R, we cannot recommend that data synthetized with this procedure be shared broadly. Formal differential privacy would provide mathematical bounds on privacy loss; absent such guarantees, empirical evaluation for reidentification risk would be necessary but insufficient to ensure privacy. We, therefore, recommend that researchers using our rote workflow do so for the purposes of internal validation, reproducibility of methodologies, and aiding in balancing otherwise unbalanced groups rather than external data sharing.

A final consideration is the computation time required to conduct the procedure we have described here. While the source TTS dataset is small, complete, and with simple univariate distributions and bivariate relationships, a GAN with a larger, more complex dataset – with more variables, more cases, more nuanced relationships, or some combination therein – will take longer to fit. Generating the dataset as we have described took approximately forty seconds of computation time on a Dell Precision 3560 with an 11^th^ Generation Intel Core i5 processor and 16 GB of RAM, but when used on the 1020 generated cases, this same process took eleven minutes. This computational requirement should be taken into consideration when using this methodology.

### Practical Considerations

Due to the complex nature of the GAN modeling framework (and generative ML models, broadly) and the data source-specific considerations necessary for a well-tuned GAN, we cannot provide uniform recommendations across all situations. We have provided some of the questions we found useful in implementing this methodology in Table 1, from making the decision to use a GAN to generate synthetic data through reporting results from the secondary model constructed with these data. While not an exhaustive list, the questions in Table 1 will allow the interested reader to begin determining if generative ML models and synthetic data should and could be implemented in their own work.

## Conclusion

This tutorial illustrated one workflow for enhancing reproducibility, transparency, and consistency to improve the rigor of biomedical research and aid in building a culture of transparent, reproducible, generalizable research. By the various sources of evidence presented:

- Similar univariate descriptive statistics between the synthetic and source data;
- Similar topography of the plots relating logistic regression-predicted probability of TTS diagnoses to the AHP/IHP ratio; and
- Similar model fit and accuracy measures;

we have demonstrated that not only are the results of Ahmed et al.’s study [9] consistent, but that the same prediction model applied to a novel analogous dataset yielded these comparable results. Additionally, in sharing the R script and synthetic dataset to illustrate the workflow we have described, we are further demonstrating transparency and ethical sharing of data. While AI tools like ChatGPT allow for generation of synthetic data from original data sources, these tools are neither secure nor tailored to the individual data source and research questions. Consequently, the implementation of GANs may allow for the broader integration of synthetic data into processes promoting internal and external replication and validation, methodological transparency, and generalizability.

## Data Availability

Source data described in this manuscript are confidential and cannot be shared. All other data and code contained in this manuscript are available via the following GitHub repository: https://github.com/UKBiostatCIRCL/Public-Code/tree/main/GAN%20Tutorial%20Manuscript.
